# Galangin delivered by retinoic acid-modified nanoparticles targeted hepatic stellate cells for the treatment of hepatic fibrosis†

**DOI:** 10.1039/d2ra07561j

**Published:** 2023-04-06

**Authors:** Yuanguo Xiong, Bing Wu, Xianxi Guo, Dong Shi, Hao Xia, Hanlin Xu, Xiaoxiong Liu

**Affiliations:** a Department of Pharmacy, Renmin Hospital of Wuhan University Wuhan 430060 China; b School of Pharmaceuticals, Hubei University of Chinese Medicine, No. 1 HuangJiahu Road West Wuhan 430065 China xhl@hbtcm.edu.cn +86 27 68890239; c Department of Cardiology, Renmin Hospital, Hubei University of Medicine Shiyan 442000 China; d Department of Cardiology, Renmin Hospital of Wuhan University Jiefang Road 238 Wuhan 430060 China xiahao1966@163.com liuxx@whu.edu.cn +86 27 88041911 +86 27 88041911; e Cardiovascular Research Institute, Wuhan University Jiefang Road 238 Wuhan 430060 China; f Hubei Key Laboratory of Cardiology Wuhan 430060 China

## Abstract

Hepatic fibrosis (HF) is a chronic hepatic pathological process induced by various liver injuries, with few available therapies. Previous research studies revealed that HF is characterized by the accumulation of excess extracellular matrix in the liver, mainly overexpressed by activated hepatic stellate cells (HSC). Therefore, HSC have been targeted in clinical trials for the management of HF. The aim of the present study was to develop an anti-HF drug delivery system with acrylic resin (Eudragit® RS100, Eud RS100) nanoparticles (NPs) through modification by retinoic acid (RA), modified for binding the retinol-binding protein reporter (RBPR) in HSC. Galangin (GA), is a multiple effects flavonoid which has demonstrated an anti-HF effect in our previous studies. In this study, GA was utilized for the treatment of HF. The results revealed that the NPs were well formed (diameter: 70 nm), spherical in shape, and exhibited uniform distribution and a high encapsulation efficiency. Moreover, a prominent controlled release effect and a significant increase in bioavailability was observed following the encapsulation of GA in NPs. These findings indicated that the limitation of low bioavailability due to the hydrophobic feature of GA was overcome. Furthermore, the pharmacodynamics studies demonstrated that NPs could drastically influence the anti-HF effects of GA after modification with retinoic acid. The results of the present study suggested that retinoic acid-modified GA NPs represent a promising candidate in the development of an anti-HF drug delivery system for the treatment of HF.

## Abstract 2

A. The preparation of Retinoic acid modified Galangin-Eud RS100 Nanoparticles and reversed hepatic fibrosis; B. the anti-HF effect of RA-GA-NPs in liver cells; C. RA-GA-NPs bind to the Retinol-Binding Protein Reporter in HSCs *via* the receptor-ligand interaction.

## Graphical abstract

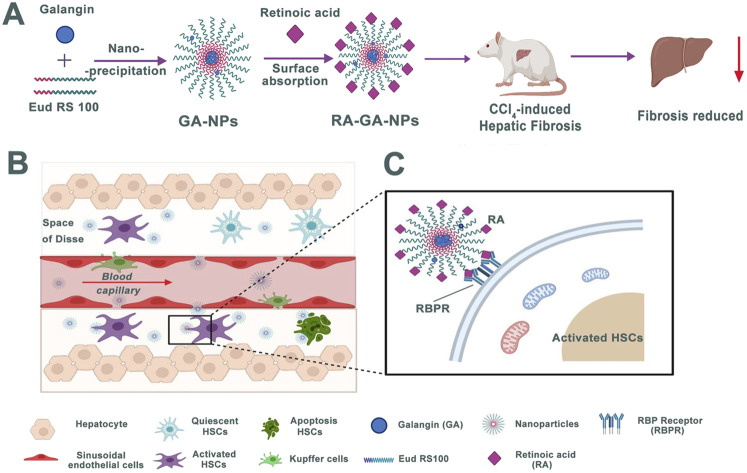

## 1 Introduction

Hepatic fibrosis (HF) is a chronic pathological tissue and cellular process associated with chronic hepatic diseases, such as viral infection, alcoholic, non-alcoholic, and drug-induced hepatitis.^1,2^ If left untreated, this condition can progress to hepatic cirrhosis and carcinoma.^3^ It has been shown that HF could be treated to some extent and reversed; thus, anti-HF research has attracted considerable attention. However, currently, there is no approved treatment for clinical trials. HF is characterized by the accumulation of excess extracellular matrix (ECM) in the liver, mainly expressed by activated hepatic stellate cells (HSC) in the perisinusoidal space of Disse.^4,5^ Under normal physiological conditions, HSC are in a quiescent state (termed quiescent HSC [qHSC]) and function as vitamin A-storing cells.^6^ However, pro-fibrotic adipocytokines can induce the activation, proliferation, and differentiation of qHSC into myofibroblast-like cells (also termed activated HSC [aHSC]). As a result, they could overexpress ECM proteins and break the ECM metabolism balance. Thus, the excess ECM accumulates intercellularly, thereby accelerating and aggravating the development of HF. Therefore, preventing HSC activation and proliferation, as well as inducing the apoptosis of HSC, could assist in managing HF. Targeting HSC could be a principal therapy in the management of HF in clinical trials.^7^

Several drug delivery systems (DDS) targeting HSC have been developed and validated in previous studies. Unmodified liposomes are passively targeting HSC through the reticuloendothelial system (RES), and have been used as a delivery vector of phenyl ethanol glycoside (CPhG) to significantly reduce HF.^8^ This is achieved by inhibiting the activation of hematopoietic stem cells and increasing the apoptosis of liver HSC. However, these liposomes, which have a particle size <200 nm and accumulate in liver cells, lack selectivity. In previous research, the mannose-6-phosphate/insulin-like growth factor II (M6P/IGFII) receptor – one of the platelet derived growth factor β (PDGFβ) receptors external to HSC – bound the rhodopsin (RHO) kinase inhibitor Y27632 to a albumin-based drug vector (PPB-MSA). This process effectively reduced parameters associated with fibrosis *in vitro* and *in vivo*.^9^ Nevertheless, to the best of our knowledge, M6P/IGFII receptors are located in HSC, as well as in endothelial and Kupffer cells; hence the specificity of M6P/IGFII receptors is doubtful. A pentapeptide for integrin αvβ3 on HSC (cRGDyK [cyclo Arg-Gly-Asp-DTyr-Lys]) modified vismodegib liposomes to improve selectivity, inhibit aHSC instead of activating qHSC, and alleviate HF.^10^ The application of particular proteins and ligands improves the anti-HF effect, while the production of pharmaceutical preparations involving the synthesis of specific proteins may be more difficult. Therefore, the discovery of a highly effective, specific, and inexpensive method for targeting HSC is required to increase the efficacy of anti-HF treatment.

**Fig. 1:**
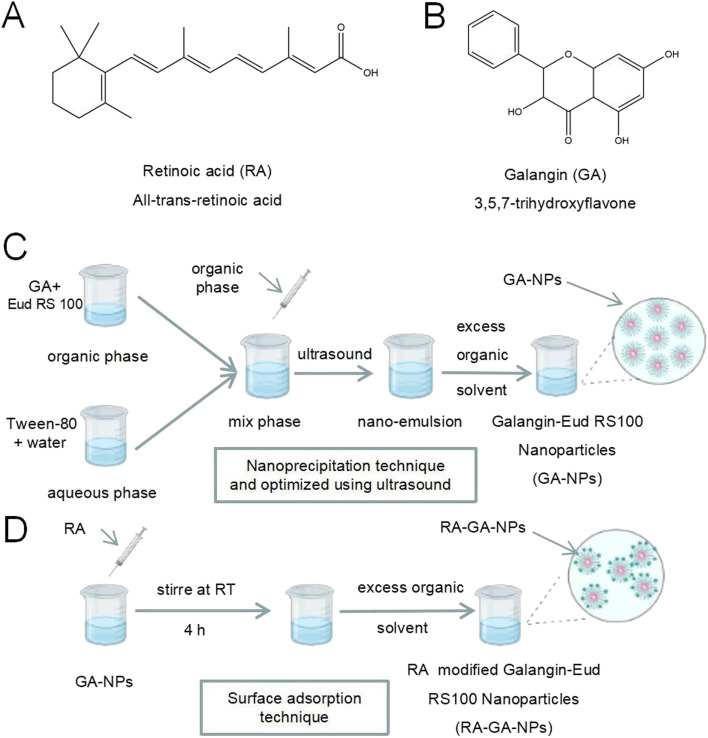
The scheme of nanoparticles formulation process. (A) The chemical structure of retinoic acid; (B) the chemical structure of galangin; (C) the preparation of GA-NPs; (D) the preparation of RA-GA-NPs.

Retinoic acid (RA) (Fig. 1A) is a bio-active form of vitamin A when it metabolites in the liver, and may be useful in targeting HSC because it specifically binds to the retinol-binding protein reporter (RBPR).^11–13^ In earlier studies of RA-modified biocompatible micelles and RA-scaffold lipid material-based nanoparticles (NPs), RA exhibited higher selectivity and specificity in targeting HSC than other ligands.^14–16^ In different rat models, RA-conjugated siRNAgp46 liposomes addressing liver collagen deposition demonstrated the remarkable potential of RA in HSC-labeled DDS.^17^ In addition, RA is a natural active, abundant, and cost-effective molecule, thereby avoiding complex and expensive artificial synthesis. Taken together, these results suggest that RA is an appropriate molecule for the development of an anti-fibrosis DDS.

Galangin (GA) (Fig. 1B) (3,5,7-trihydroxyflavone) is a natural polyphenolic compound extracted from the rhizome of *Alpinia officinarum* Hance. It has been used as a traditional polyphenolic compound medicine in South Asia for centuries. Previous studies have reported various pharmacological properties of GA, such as anti-inflammatory, anti-oxidant, anti-tumor, anti-allergy, and anti-Alzheimer's disease activity.^18–21^ In addition, GA exerts a protective effect against alcohol-induced liver damage in mice by alleviating oxidative stress and lipid peroxidation.^22,23^ Our earlier studies also revealed that GA shows anti-fibrotic activity in LX-2 cells by inhibiting the PI3K/Akt pathway and its downstream pathways.^24^ However, after oral administration, GA exhibits low bioavailability due to its water-soluble hydrophobicity; this limits its clinical application.

NPs and nanotechnology are multifunction tools in pharmaceuticals, offering practical solutions for unsuitable components, enhancing bioavailability, reducing toxicity, and improving efficacy.^25–27^ Eudragit® RS100 (Eud RS100), a type of acrylic resin, contains quaternary ammonium groups (4.5–6.8%) which provide a positive charge on the surface.^28^ Through charge adsorption, NPs with Eud RS100 can interact with negatively charged bioactive molecules or cellular target tissues, thereby achieving different targeting functions.^29^ In addition, Eud RS100 shows water-insolubility and swelling properties at physiological pH, thereby offering advantages in terms of drug dispersion and as a delivery vector for different drugs.^30^ Based on these features, Eud RS100 could be utilized for the development of GA DDS.

The aim of the present study was to develop an HF-targeting DDS for enhancing the anti-fibrotic effect of GA following oral administration. Therefore, Eud RS100 was adopted as a polymer for the incorporation of GA (galangin-Eudragit® RS100 nanoparticles [GA-NPs]). Moreover, RA was modified on the surface of GA-NPs to specifically target HF (retinoic acid-modified galangin-Eudragit® RS100 nanoparticles [RA-GA-NPs]). Subsequently, the physicochemical characteristics of NPs were assessed to evaluate the preparation method of GA-NPs. Moreover, the anti-fibrotic effect of GA-NPs was investigated *in vitro* to serve as a reference for future pharmaceutical research and clinical applications.

## 2 Materials and methods

### 2.1 Materials and reagents

GA (content 98%, batch no.: PS020516) was purchased from Chengdu Pusi Biotechnology Co., Ltd (Chengdu, China). RA (content 98%, batch no.: K1905021) was obtained from Aladdin Pharmaceutical (Shanghai, China). Eud RS100 was provided by Roehm Chemical Co., Ltd (Shanghai, China) and Tween-80 was provided by China National Pharmaceutical Group Co., Ltd (Wuhan, China). Indocyanine green was obtained from Macklin Biochemical Co., Ltd (Shanghai, China). Methyl alcohol (high-performance liquid chromatography [HPLC] grade) and acetonitrile (HPLC grade) were purchased from Macklin Biochemical Co., Ltd (Shanghai, China). The alanine aminotransferase (ALT), aspartate aminotransferase (AST), and hepatic hydroxyproline (Hyp) testing kits were obtained from the Nanjing Jiancheng Bioengineering Institute (Nanjing, China). Hyaluronic acid (HA), laminin (LN), collagen type IV (Col IV), and procollagen III (PC III) enzyme linked immunosorbent assay (ELISA) kits were purchased from BioSharp Co., Ltd (Wuhan, China). The deionized purified water was prepared in the laboratory, and other reagents were of analytical grade.

### 2.2 Preparation of NPs

GA-NPs were prepared with the nanoprecipitation technique and optimized using ultrasound (Fig. 1C).^31^ Briefly, GA (1 mg) and Eud RS100 (150 mg) were dissolved in 2 mL of methanol, forming an organic phase at room temperature, and mixed under ultrasonic conditions for 15 min. Next, the organic phase was slowly added to 4 mL of aqueous phase formed using deionized purified water with Tween-80 (1.0%, volume : volume) under gentle stirring. Thereafter, homogenization was conducted with ultrasound (10 min, 0.1 s on, and 0.2 s off) through gentle continuous mechanical stirring for another 30 min. Finally, the excess organic solvent was removed under rotary vacuum evaporation at 50 °C, and the GA-NP suspension was obtained. Blank-NPs were prepared through the same process, without dissolving GA in methanol. All samples were prepared in duplicate.

RA-GA-NPs were prepared using the surface adsorption technique (Fig. 1D).^32^ Briefly, RA (100 mg) dissolved in 1 mL of methanol was added to GA-NPs (10 mL) and stirred at room temperature for 4 h. After removing organic solvents with rotary vacuum evaporation at 50 °C, the RA-GA-NP suspension was obtained. RA-GA-NPs were characterized based on their mean particle size, polydispersity index (PDI), and zeta potential. Blank-RA-NPs were prepared through the same process as that for Blank-NPs. All samples were prepared in duplicate.

### 2.3 Characterization of NPs

The mean particle size, PDI, and zeta potential of the NPs were evaluated using a laser particle size instrument (Zetasizer ZS90; Malvern Instruments Ltd, Malvern, Worcestershire, UK). GA content in the NPs was evaluated using the HPLC method (ESI Fig. S1†); this experiment was performed in triplicate. Suspensions of NPs (1.0 mL) were ultracentrifuged at 15 000 rpm for 30 min at 4 °C. Subsequently, NPs were collected, washed thrice, and re-dispersed in 1 mL of deionized purified water. Next, they were mixed with 5.0 mL of acetonitrile for emulsion breaking, and measured using the HPLC method. The amount of encapsulated drug loading (DL) was determined using eqn (I):I
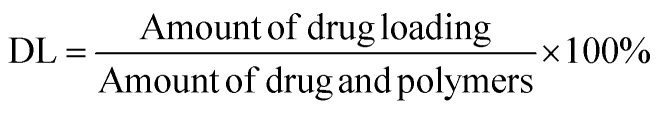


Suspensions of NPs (1.0 mL) were directly added to 5.0 mL of acetonitrile for emulsion breaking and measured using the HPLC method to determine the amount of total drug loading. This experiment was performed in triplicate. The encapsulation efficiency (EE) was calculated using eqn (II):II
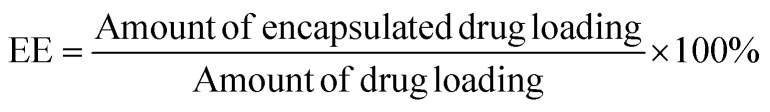


### 2.4 Transmission electron microscopy (TEM)

The morphology of NPs was determined using TEM (JEOL Ltd, Akishima, Japan). Initially, the sample was negatively stained with 2% (weight : volume) phosphotungstic acid, added to a carbon-coated copper meshwork, and dried at room temperature. Subsequently, the sample was analyzed using TEM at an acceleration voltage of 200 kV.

### 2.5 Fourier-transform infrared spectroscopy (FTIR)

The lyophilized NPs powder was prepared using the vacuum freeze-drying method for FTIR. Briefly, 5.0 mL of NPs were added to a vial. The vial was pre-frozen for 24 h at −74 °C in an ultralow temperature freezer and maintained in the lyophilizer for another 48 h to obtain the lyophilized NPs powders. FTIR spectra of the co-amorphous combinations were analyzed. Their corresponding crystalline and amorphous drugs were obtained using the FTIR spectrometer (Thermo Scientific, Waltham, MA, USA) over the wavenumber range of 4000–400 cm^−1^, with a resolution of 4 cm^−1^ and an accumulation of 64 scans.

### 2.6 Differential scanning calorimetry (DSC)

DSC (Hitachi High-Tech Ltd, Tokyo, Japan) was carried out for the thermal analysis of pure compounds and lyophilized NPs powders. Briefly, each sample (2 mg) was placed onto standard aluminum pans and sealed; an empty pan was used as a reference. DSC scans were performed at a heating rate of 10 °C per min for a temperature range of 40–400 °C under a nitrogen atmosphere. Aluminum oxide was used as the standard material to calibrate the temperature and the energy scale of the DSC instrument.

### 2.7 *In vitro* release studies

NPs (1 mL) were put into a dialysis bag (molecular weight cut-off: 8000–12 000; Merck KGaA, Darmstadt, Germany). Subsequently, the dialysis bag was placed in Erlenmeyer flasks containing 100 mL of dissolution medium (pH 7.4 and phosphate buffer containing 0.5% Tween-80 to meet the sink condition) and stirred at 120 rpm at 37 ± 1 °C. Aliquots (1 mL) were obtained from the supernatant at predetermined time intervals (*i.e.*, 0.25, 0.5, 0.75, 1, 2, 4, 6, 8, 10, 12, 24, 48, 72, and 96 h). An equal volume of fresh medium was added to replace the extracted supernatant. Drug concentrations in the dissolution medium were measured using the HPLC method. The control, containing the same amount of GA with that contained in Blank-NPs, was prepared and analyzed through the same process.

### 2.8 Pharmacokinetics

All animal procedures were approved by the Animal Care and Use Committee of the Renmin Hospital of Wuhan University (Laboratory Animal Welfare and Ethics Committee [Institutional Animal Care and Use Committee] Issue No.: WDRM NO.20200501). Eighteen 6 weeks-old Sprague–Dawley rats (weight: 200 ± 20 g; male: *n* = 9; female: *n* = 9) were randomly divided into three groups (*n* = 6 per group). Free GA, GA-NPs, and RA-GA-NPs were orally administered (GA concentration: 50 mg kg^−1^). Subsequently, 200 μL of blood were collected at predetermined time points (*i.e.*, 0.5, 1, 2, 4, 6, 8, 10, 12, and 24 h). The concentrations of GA in plasma were determined by HPLC. Plasma samples (200 μL) were extracted using 200 μL of acetonitrile, vortexed for 10 min, and centrifuged at 5000 rpm for 5 min. Thereafter, the supernatant was collected and filtered using a 0.45 μm nylon 66 membrane. Finally, each sample (20 μL) was injected into the HPLC system.

### 2.9 Small animal imaging

The body distribution of NPs was analyzed through small animal imaging. Eighteen 6 weeks-old C57BL/6 mice (weight: 20 ± 2 g; male: *n* = 9; female: *n* = 9) were randomly divided into three separate groups (*n* = 6 per group). Indocyanine green and free GA solution, indocyanine green loaded GA-NPs, and indocyanine green-loaded RA-GA-NPs (indocyanine green concentration: 1 mg kg^−1^) were orally administered. Following anesthesia with trifluoroethane, the immunofluorescence signals were captured with a cryostat microtome (Leica, Wetzlar, Germany) at 0.5, 1, 1.5, 2, 3, and 4 h.

### 2.10 Anti-HF effect *in vivo*

Forty 6 weeks-old male C57BL/6 mice (weight: 20 ± 2 g; male: *n* = 20; female: *n* = 20) were randomly divided into five groups (*n* = 8 per group): control; tetrachloromethane (CCl_4_)-treated; free GA; GA-NPs; and RA-GA-NPs. The HF model was induced by intraperitoneal injection with 10% CCl_4_ (diluted in olive oil, 5 mL per kg body weight) thrice per week for 4 consecutive weeks.^33–35^ The control mice received an intraperitoneal injection with an equal volume of olive oil. The mice in all treatment groups received an injection with CCl_4_ for 4 weeks, as well as free GA, GA-NPs, or RA-GA-NPs (20 mg kg^−1^) through oral administration once daily for 4 weeks; the dose of GA was based on previous reports.^36,37^ All mice were sacrificed 12 h after the last administration, and blood specimens were collected through retro-orbital bleeding. Serum was subsequently separated by centrifugation at 3500 rpm for 15 min at 4 °C and stored at −20 °C. The liver tissues were rapidly dissected, immediately washed with cold phosphate-buffered saline, frozen in liquid nitrogen, and stored at −80 °C or fixed with 4% paraformaldehyde.

Hematoxylin–eosin staining (H&E) is typically used to discriminate collagen fibers from tissues on histological slides. Therefore, liver sections stained with Picro Sirius Red (PSR) were examined microscopically (Olympus, Tokyo, Japan) to determine collagen deposition. Five different fields were randomly observed in each slice, and three slices were selected from each group. An experienced pathologist blinded to the study protocol performed all histological examinations. The levels of ALT, AST, and Hyp were determined using detection kits, respectively, according to the instructions provided by the manufacturer. In addition, the serum levels of markers, HA, LN, PC III, and Col IV were determined using ELISA kits based on the respective protocols.

### 2.11 Statistical analysis

All data are presented as the mean ± standard error of the mean, and all experiments were repeated in triplicates. Data were analyzed using Student's *t*-test and one-way analysis of variance with GraphPad Prism version 6 (GraphPad Software, La Jolla, CA, USA). The *p*-values < 0.05 denoted statistically significant differences.

## 3 Results and discussions

### 3.1 Characterization of NPs

**Fig. 2:**
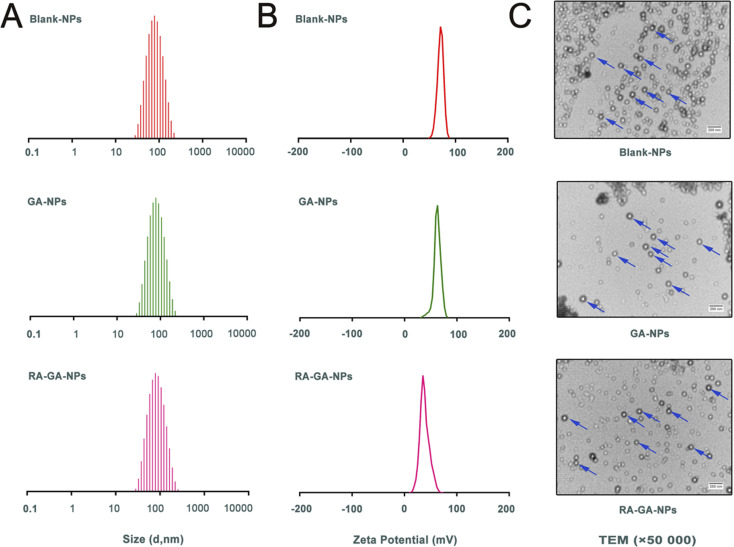
The physical characterization of nanoparticles. (A) the adequate particle size of nanoparticles; (B) the zeta potential of nanoparticles; (C) the transmission electron microscope (TEM) photograph of nanoparticles.

The Blank-NPs, GA-NPs, and RA-GA-NPs showed adequate particle size with average diameter of 69.85 ± 0.478, 71.66 ± 0.410, and 73.55 ± 0.726 nm, respectively; PDI of 0.199 ± 0.003, 0.187 ± 0.005, and 0.227 ± 0.010, respectively; and zeta potential of 62.3 ± 3.71, 65.1 ± 2.04, and 41.5 ± 0.78 mV, respectively (Fig. 2A and B). The EE of GA-NPs and RA-GA-NPs was 97.33 ± 1.02% and 95.04 ± 1.37%, respectively. The DL of GA-NPs and RA-GA-NPs was 0.65 ± 0.0056% and 0.63 ± 0.0055%, respectively. The capacity of GA-NPs and RA-GA-NPs was 242.78 ± 2.10 and 238.61 ± 2.11 mg, respectively. TEM was used to visualize the NPs in more detail. Fig. 2C shows the surface morphology, depicting NPs with a diameter of 70 nm, spherical shape, and uniform distribution.

In recent decades, nanotechnology has been recognized as a multi-functional tool in pharmaceutical research. This is because it provides effective solutions to ill-posed or ill-adapted components by enhancing their bioavailability, reducing their toxicity, and increasing their efficacy.^38–40^ Moreover, nanotechnology allows the development of desired ligands for targeting purposes.^41^ In this study, a two-pronged strategy was adopted to ensure specificity. Firstly, GA-NPs were prepared to overcome the limitations of the physicochemical properties of GA. Uncoated NPs loaded with GA were produced by the classical nanoprecipitation method and optimized with sonication.^42^ The NPs showed appropriate sizes (<100 nm), a narrow distribution with a PDI <0.25, which could easily taken up by RES for passive targeting of the liver.^43^ The EE in NPs was high (>95%), as GA is a hydrophobic and water-insoluble drug. This finding indicated that the nanoprecipitation technique offers a feasible strategy for the efficient incorporation of GA into NPs. In addition, the NPs exhibited low levels of SD and reliable repeatability. The results showed that the combination of nanoprecipitation and ultrasonication is an appropriate method for preparing NPs with favorable particle size and PDI. Secondly, the delivery of GA specifically to HSC in the liver was performed by RA-GA-NPs. Owing to Eud RS100, a well-established cationic nature polymer, the NPs showed positive values, indicating the potential to adhere to negatively charged ligands (*i.e.*, DNA and protein).^44–46^ After binding with RA, the zeta potential of NPs was significantly decreased, indicating that charge neutralization occurred on the surface of NPs after the adsorption of negative RA. The drug–polymer ratio was 1 : 150; hence, NPs had a lower DL than the galactose bound to F68-GA micelles (4.35 ± 0.55%) and GA/β-Cyclodextrin inclusion complex (2.41%).^47,48^ Based on our previous study, GA is highly effective against HF, as the inhibition of aHSC *in vitro* was initiated at the concentration of 6 μg mL^−1^.^24^ The capacity of GA-NPs and RA-GA-NPs was approximately 240 mg. This capacity could meet the requirement for drug administration and avoid toxicity induced by an overdose. In addition, NPs showed reliable stability over 6 months, as a zeta potential >±30 mV contributes to the physical stability of the colloidal system (ESI Fig. S2†).^49^ Finally, we demonstrated that the NPs specifically targeting HSC (RA-GA-NPs) were well formed.

### 3.2 FTIR

**Fig. 3:**
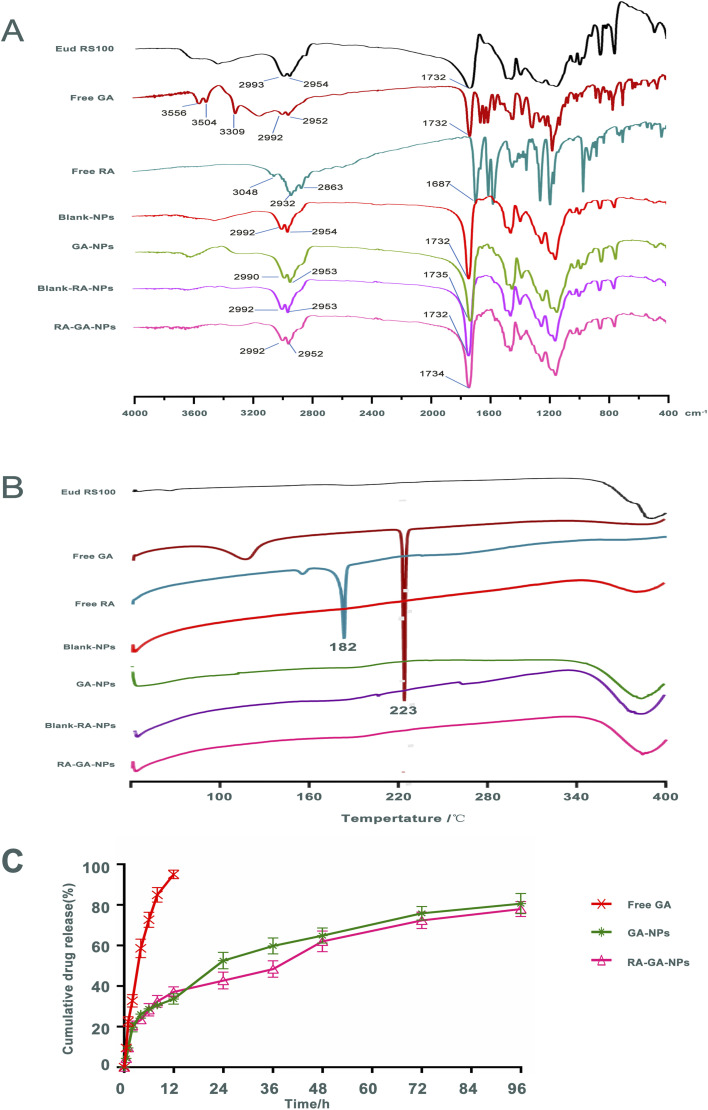
The quality control results of nanoparticles. (A) the fourier transform infrared spectroscopy (FTIR) of nanoparticles and its ingredients. (B) the differential scanning calorimetry (DSC) of nanoparticles and its ingredients; (C) the release profiles of free GA, GA-NPs and RA-GA-NPs *in vitro* (*n* = 3).

Fig. 3A shows the FTIR spectra of Eud RS100, free GA, free RA, Blank-NPs, GA-NPs, Blank-RA-NPs, and RA-GA-NPs. Infrared spectroscopy of the Eud RS100 revealed typical polymer bands, including bands at 2993 and 2954 cm^−1^ for –CH, and 1732 cm^−1^ for C=O. The infrared spectra of free GA indicate characteristic drug bands, including bands at 3556, 3504, and 3309 cm^−1^ for –OH, 2992 and 2952 cm^−1^ for –CH, and 1732 cm^−1^ for C=O. Free RA demonstrated typical polymer peaks, including peaks at 3048 cm^−1^ for –OH, 2932 and 2863 cm^−1^ for –CH, and 1687 cm^−1^ for C=O. The infrared spectrum of Blank-NPs displayed similar bands to those observed in the FTIR spectra of the Eud RS100, with bands at 2992 and 2954 cm^−1^ for –CH, and 1732 cm^−1^ for C=O. The FTIR spectrum of GA-NPs revealed corresponding peaks to these compounds, including bands at 2992 and 2954 cm^−1^ for –CH, and 1735 cm^−1^ for C=O. The infrared spectrum of Blank-RA-NPs and RA-GA-NPs displayed, the majority of existing bands in the FTIR spectrum of the GA-NPs as the typical band at 1735 cm^−1^ (C=O) was shown. Comparison of the FTIR spectrum of drug and NPs, showed that there were no distinctive changes in GA and RA when in contact with polymers, indicating that Eud RS100 was not involved in intermolecular interaction.^50^ Similar results were reported in earlier studies on the preparation of Eud RS100 NPs by nanoprecipitation.^51,52^ However, there were absent –OH bands around 3000 cm^−1^ of GA and RA in NPs, due to the intermolecular hydrogen bonding between GA and RA with Eud RS100. These results indicated that the chemical stability of the NPs depends on van der Waals forces rather than covalent bonds.^53^

### 3.3 DSC

DSC was used to determine the physicochemical characteristics of NPs. Fig. 3B shows the DSC thermograms of the Eud RS100, free GA, free RA, Blank-NPs, GA-NPs, Blank-RA-NPs, and RA-GA-NPs. The free GA exhibited a large endothermic peak at 223 °C, corresponded to its melting point; this indicated that the intact GA was in a crystalline anhydrous state. Similar results were obtained for free RA, which displayed a sharp endothermic peak at approximately 182 °C. The Eud RS100, Blank-NPs, GA-NPs, Blank-RA-NPs, and RA-GA-NPs did not show a sharp endothermic peak at 40–400 °C. The lack of a peak of GA or RA in the DSC profiles might be due to the decreased crystallinity in the formulations and/or drug solvation in the amorphous carrier, as well as solid-state interaction induced by heating.^54^ Therefore, we could conclude that both GA and RA in Eud RS100 were captured by physical mixing rather than chemical reaction.

### 3.4 *In vitro* release studies

Fig. 3C depicts the GA profile from NPs during *in vitro* release. Within 2 h, approximately 20% of the GA was burst released. Thereafter, the rate of drug release was reduced, and approximately 80% of the total drug was released by 96 h. The cumulative release of the GA-NPs curves was consistent with the Higuchi equation and expressed by eqn (III):

III *Q* = 0.0467*t*^1/2^ + 0.0607*r* = 0.9858

The cumulative release of the RA-GA-NPs curves was consistent with the Higuchi equation and expressed by eqn (IV):

IV *Q* = 0.043*t*^1/2^ + 0.0656*r* = 0.9856

The release rate of GA in NPs was significantly slower than that of free GA, which indicated that NPs containing Eud RS100 exerted a sustained release effect *in vitro*. Eud RS100, as a water-insoluble carrier, was able to control drug release in NPs. All NPs showed slower drug release in comparison with the intact drug. The GA-loaded NPs exhibited a burst release within 2 h, which was attributed to the drug loaded on the surface of NPs. A similar burst release was observed in desloratadine- and naproxen-Eud RS100 NPs prepared by emulsification solvent evaporation.^55,56^ It was expected that NPs would show an initial burst release, as a result of the adsorption of the drug on the surface.^57^ Drug encapsulation on the superficial layers of the particles due to the smaller size of NPs could explain the rapid release, while the diffusion and dissolution of the drug from inner layers were responsible for the plateau phase.^58^

The cumulative release curves of GA-NPs and RA-GA-NPs were consistent with the Higuchi equation, indicating that the drug diffused from the polymer matrix under physiological conditions.^51^ Similar release behavior *in vitro* was also observed for Eud RS100 nanocapsules and Arg-Gly-Asp-labeled (RGD-labeled) polymersomes loaded with clotrimazole and oxymatrine, respectively.^59,60^ However, there was no statistical difference between the release patterns of GA-NPs and RA-GA-NPs due to their similitude in terms of physical characteristics, such as NPs size, which influenced the dissolution and diffusion rate of GA.^54^

### 3.5 Pharmacokinetics

**Fig. 4:**
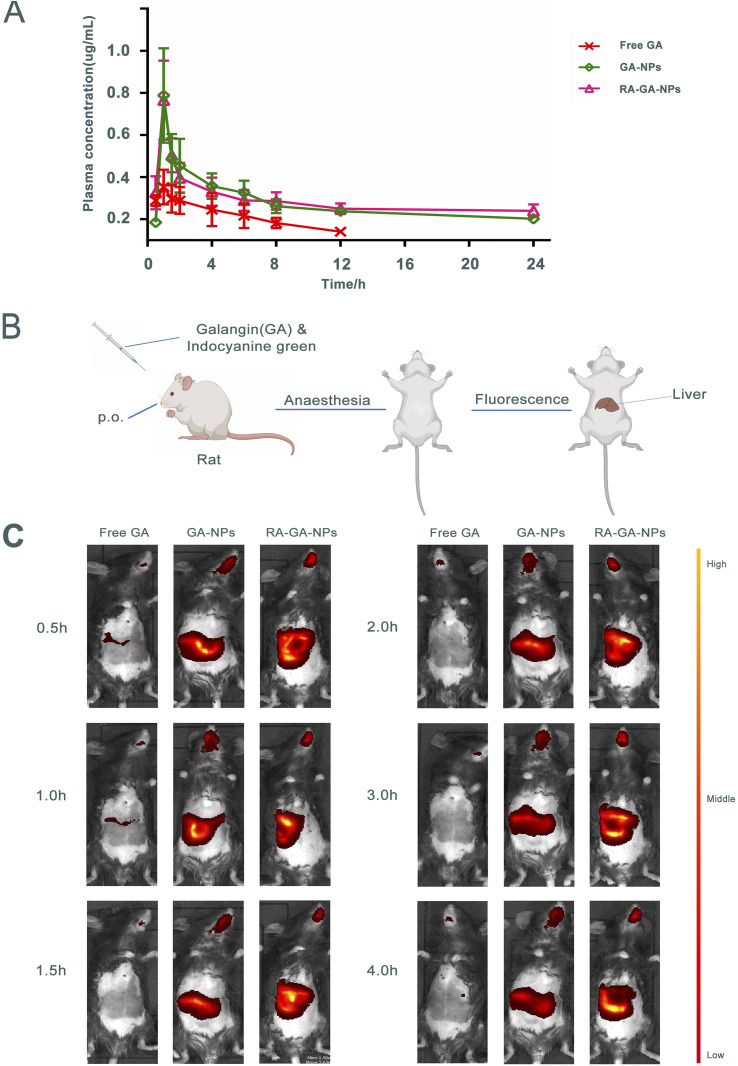
The absorption and distribution of nanoparticles *in vivo*. (A) the concentration–time curve of GA in plasma after single oral administration of free GA, GA-NPs and RA-GA-NPs (*n* = 6); (B) the illustration of small animal imaging in mice; (C) the representative images of the distribution of GA in mice after single oral administration of free GA, GA-NPs and RA-GA-NPs in 4 h (*n* = 6).

The mean plasma concentration–time curve after the oral administration of GA is shown in Fig. 4A. Briefly, the peak plasma concentration of both GA forms was achieved within 1 h. The concentrations of GA-NPs and RA-GA-NPs were significantly higher than that of free GA, which was not the case for GA-NPs and RA-GA-NPs respectively. Serum concentrations of free GA were detected only for up to 12 h. However, serum drug concentrations were maintained for 24 h after the oral administration of GA-NPs and RA-GA-NPs.

Table 1 demonstrates the important pharmacokinetic parameters of GA in mouse serum. Compared with free GA, both GA-NPs and RA-GA-NPs were linked to a 1.95- and 2.04-fold increase in the elimination half-life (*t*_1/2_) of GA, respectively (*p* < 0.01). Similarly, the serum levels of GA (area under the concentration–time curve, AUC) from GA-NPs and RA-GA-NPs exhibited a 2.55- and 2.65-fold increase, respectively, compared with those of free GA (*p* < 0.01). Compared with the free drug solution, the GA plasma clearance rate for GA-NPs and RA-GA-NPs demonstrated a significant decrease (*i.e.*, 2.59- and 2.71-fold reduction, respectively) (*p* < 0.01). Both NP formulations lowered the apparent volume of distribution (*p* < 0.05) of GA. The volume of distribution of GA decreased from 20.98 mL in free GA to 15.75 mL and 15.82 mL in GA-NPs and RA-GA-NPs, respectively. Furthermore, a statistically significant difference (*p* < 0.05) in the mean residence time of GA was observed between the treatment groups. The mean residence time exhibited a 1.94- and 2.04-fold increase for GA-NPs and RA-GA-NPs, respectively, compared with that recorded for free GA.

**Table 1:** The pharmacokinetic parameters of GA following oral administration with free GA, GA-NPs and RA-GA-NPs (*n* = 6)^a^^,^^b^

Pharmacokinetic parameters	Free GA	GA-NPs	RA-GA-NPs
AUC [(μg mL^−1^)·h]	2.58 ± 0.47	6.57 ± 0.69^*aaa*^	6.83 ± 0.65^*aaa*^
AUMC (μg mL^−1^)	13.49 ± 2.16	66.96 ± 5.19^*aaa*^	73.38 ± 8.26^*aaa*^
MRT (h)	5.25 ± 0.16	10.23 ± 0.51^*aaa*^	10.73 ± 0.48^*aaa*^
*t*_1/2_ (h)	3.64 ± 0.11	7.09 ± 0.35^*aaa*^	7.44 ± 0.33^*aaa*^
CL [(mg kg^−1^)/h(μg mL^−1^)]	3.99 ± 0.73	1.54 ± 0.15^*aaa*^	1.47 ± 0.14^*aaa*^
*V*_d_ (L kg^−1^)	20.98 ± 4.22	15.75 ± 2.18^*a*^	15.85 ± 1.49^*a*^

a Abbreviations: GA, galangin; GA-NPs, Galangin-Eud RS100 nanoparticles; RA-GA-NPs, Retinoic acid modified Galangin-Eud RS100 nanoparticles; AUC, area under the concentration–time curve; AUMC, area under the first moment concentration–time curve; MRT, mean residence time; *t*_1/2_, elimination half life; CL, plasma clearance; *V*_d_, apparent volume of distribution.

b Note: ^*a*^ means *p* < 0.05 *vs.* the free GA group. ^*aa*^ means *p* < 0.01 *vs.* the free GA group. ^*aaa*^ means *p* < 0.001 *vs.* the free GA group.

The results showed that, compared with free GA, NPs could significantly improve the absorption of GA and exerted a positive and sustained release effect. Particle size should respond to gastrointestinal absorption and the underlying mechanism of NP absorption. The NPs of smaller size could greatly increase the surface area of the absorbing tissue, enhance the permeability of the gastrointestinal membrane, prolong the residence time, and promote drug absorption by interacting with the gastrointestinal wall.^61^ The optimum particle size ranges between 10 and 100 nm, and is considered a key feature as larger particles are more easily absorbed and transported by intestinal cells.^62^ Earlier studies reported that only 7.8% of 50 nm and 0.8% of 100 nm NPs in total numbers accounted for 34% and 26% of the total oral absorption, respectively.^63^ In addition, NPs of smaller size could evade RES clearance by lymphatic transport to achieve a longer retention time in blood circulation.^64^ Of note, there was no significant difference in pharmacokinetics between GA-NPs and RA-GA-NPs. This is because the absorption region of NPs may be mainly located in the membranes of the stomach and colon, which lack specific RA-targeting receptors.

### 3.6 Small animal imaging

The mice received indocyanine green/free GA, indocyanine green-loaded GA-NPs, and indocyanine green-loaded RA-GA-NPs through oral administration. The highest fluorescence intensity in the liver was observed for indocyanine green-loaded RA-GA-NPs, followed by indocyanine green-loaded GA-NPs, and indocyanine green/free GA (Fig. 4B and C). These results showed that the fluorescence intensity of the target region was significantly increased by the incorporation of indocyanine green into NPs. Notably, indocyanine green-loaded RA-GA-NPs showed a significant improvement in fluorescence intensity *versus* indocyanine green loaded-GA-NPs throughout the entire study period. In addition, indocyanine green-loaded RA-GA-NPs and GA-NPs led to strong liver fluorescence for at least 4 h. In contrast, the indocyanine green/free GA was no longer detectable in the liver.

The results showed that the GA loaded in NPs was rapidly uptaken in the gastrointestinal tract and reached peak levels at 1 h, as well as the time to maximum concentration showed in pharmacokinetics studies. With the passage of time, the concentration of GA was gradually decreased. The concentration of GA was rapidly decreased within 2 h, while that of NPs remained high after 4 h. These findings may be attributed to two reasons. Firstly, the controlled release of NPs prolongs the retention time, while the addition of the drug to the polymer matrix prevents degradation in the gastrointestinal tract. Thus, both a passive target strategy with specific NPs (size: <100 nm) and active target strategy combining RA and NPs can be adopted to target HF. The fluorescence intensity of GA-NPs and RA-GA-NPs in the liver was significantly higher than that of free GA. This evidence confirmed that the NPs had successfully targeted the liver. In addition, the fluorescence intensity of RA-GA-NPs was significantly higher than that of RA-NPs, indicating that the integration of RA in NPs improved the distribution of GA in the mouse liver.

The biodistribution of NPs is affected by several service factors, such as NP size, surface charge, and ligands. Similar to cells that are smaller than the diameter of the sinusoidal fenestration of the liver, NPs of size <150 nm are able to enter into the space of Disse and interact directly with the HSC. The surface charge also alters the uptake by different types of hepatic cells, since electrostatic interactions and protein adsorption upon the cell membrane differ following the administration of NPs. Negatively charged NPs may be more appropriate for liver sinusoidal endothelial and Kupffer cells, while positively charged NPs may be more appropriate for hepatocytes.^65^ Moreover, unique ligands and their receptors can exert varied effects on circulating NPs. After reaching the site of action, NPs bind to their targets in the cell membrane, enhance the concentration of drug, and produce a pharmacological effect.^66^ In this study, the RA-GA-NPs were prepared with a suitable size for transport in the liver, a positive charge to avoid clearance by Kupffer cells, and RA for BRBP conjugate, thereby showing specific targeting of HSC *in vivo*.

### 3.7 Anti-fibrogenic effect *in vivo*

**Fig. 5:**
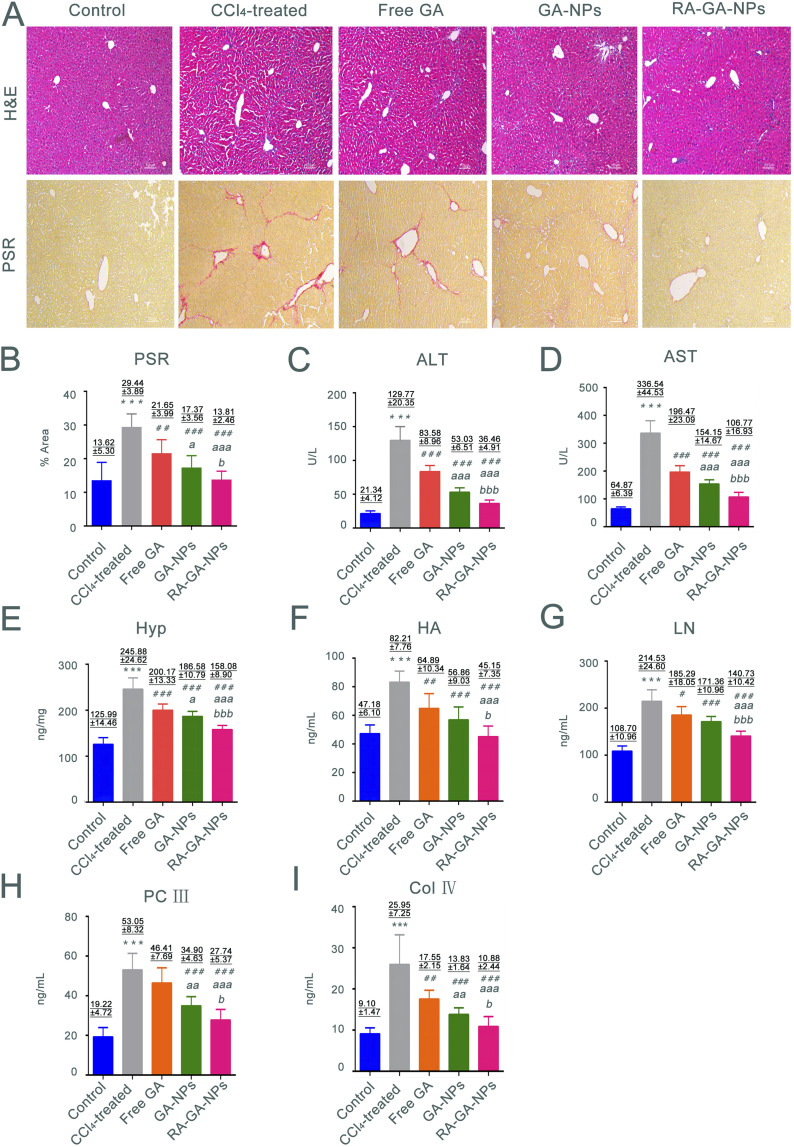
The anti-HF effects of GA in mice. (A) the representative images of H&E and PSR staining in mice after the management free GA, GA-NPs and RA-GA-NPs (20 mg kg^−1^) daily for 4 weeks (*n* = 8), the images were shown at ×100 magnification; (B) the relative positive area percent of collagen protein in PSR staining under the treatment of free GA, GA-NPs and RA-GA-NPs (*n* = 8); (C) the level of serum alanine aminotransferase (ALT) in the free GA group, GA-NPs group and RA-GA-NPs group (*n* = 8); (D) the level of serum aspartate aminotransferase (AST) in the free GA group, GA-NPs group and RA-GA-NPs group (*n* = 8); (E) the level of Hyp in mice liver after the free GA, GA-NPs and RA-GA-NPs treated for 4 weeks (*n* = 8); (F) the level of serum hyaluronic acid (HA) in the free GA group, GA-NPs group and RA-GA-NPs group (*n* = 8); (G) the level of serum laminin (LN) in the free GA group, GA-NPs group and RA-GA-NPs group (*n* = 8); (H) the level of serum procollagen III (PC III) in the free GA group, GA-NPs group and RA-GA-NPs group (*n* = 8); (I) the level of serum collagen type IV (Col IV) in the free GA group, GA-NPs group and RA-GA-NPs group (*n* = 8). Data are shown as mean ± SEM.* means *p* < 0.05 *vs.* the control group. ** means *p* < 0.01 *vs.* the control group.*** means *p* < 0.001 *vs.* the control group. ^#^means *p* < 0.05 *vs.* the CCl_4_ group. ^##^means *p* < 0.01 *vs.* the CCl_4_ group. ^###^means *p* < 0.001 *vs.* the CCl_4_ group. ^*a*^ means *p* < 0.05 *vs.* the GA solution group. ^*aa*^ means *p* < 0.01 *vs.* the GA solution group. ^*aaa*^ means *p* < 0.001 *vs.* the GA solution group. ^*b*^ means *p* < 0.05 *vs.* the GA-NPs group. ^*bb*^ means *p* < 0.01 *vs.* the GA-NPs group. ^*bbb*^ means *p* < 0.001 *vs.* the GA-NPs group.

In this study, we investigated the relationship between fibrosis and nanoparticles. This was achieved by exposing 6 weeks-old male mice to CCl_4_ to establish fibrosis. The development of fibrosis was confirmed by staining with H&E and PSR, directly marking the ECM deposited by aHSC (Fig. 5A). According to H&E staining, CCl_4_-treated mice demonstrated extensive hepatocyte disorganization and cellular infiltration. Importantly, these effects were alleviated after the administration of GA. The greatest anti-fibrotic capability was observed in RA-GA-NPs, followed by GA-NPs and free GA (*p* < 0.05). In contrast, the control animals (*i.e.*, without administration of CCl_4_) did not exhibit alterations in hepatic histology, inflammation, or liver function. The PSR staining was utilized to assess the degree of HF; the CCl_4_-treated mice had a 2.13-fold increase in staining intensity compared with the control mice (*p* < 0.001) (Fig. 5B). Furthermore, treatment with GA suppressed the marked activation of HSC, as visualized by immunofluorescence staining of liver sections with PSR, similar to H&E (*p* < 0.05).

The serum levels of AST and ALT were measured to determine whether chronic exposure to CCl_4_ influenced inflammation. Following the administration of CCl_4_ for 4 weeks, significant differences were observed in the serum levels of transaminases between the control and CCl_4_-treated mice, with considerably higher levels recorded in the latter group (*p* < 0.001) (Fig. 5C and D). The serum levels of transaminases were significantly lower in mice treated with free GA than in those treated with CCl_4_ (*p* < 0.001). The same trend was observed in the comparison between the free GA and GA-NPs groups; the ALT and AST levels in the latter group were significantly reduced (*p* < 0.001). Furthermore, the lowest serum levels of transaminases among the GA treatment groups was recorded for the RA-GA-NP group, and the difference between free GA and GA-NPs reached statistical significance (*p* < 0.001).

The Hyp content in the liver is associated with worsening of fibrosis; thus, the presence of this amino acid was analyzed to assess the degree of HF. The mice treated with CCl_4_ for 4 weeks exhibited a 1.5-fold increase in Hyp content compared with the untreated mice. Nevertheless, HF was more severe in the CCl_4_-treated mice *versus* the control mice (Fig. 5E). Furthermore, compared with the CCl_4_ group, the Hyp levels were gradually decreased in the free GA, GA-NP, and RA-GA-NP groups (*p* < 0.001).

HA, LN, PC III, and Col IV were recently identified as biomarkers in rapid blood tests for the diagnosis of HF. Exposure of mice to CCl_4_ for 4 weeks led to a significant elevation in the circulating levels of HA, LN, PC III, and Col IV. Notably, this elevation was reduced after treatment with GA-loaded NPs (*p* < 0.05) (Fig. 5F– I). RA-GA-NPs significantly decreased the serum levels of HA, LN, PC III, and Col IV compared with free GA or GA-NPs (*p* < 0.05). Free GA exerted a modest anti-fibrotic effect, whereas RA-GA-NPs drastically attenuated the biomarkers of HF (*p* < 0.01).

The classical animal model of HF induced by CCl_4_ has been used to evaluate the anti-HF effects of NPs *in vivo*. The active metabolite of CCl_4_ may cause damage to the liver through H_2_O_2_ and reactive oxygen species, altering the levels of hepatic enzymes and markers of HF.^67–69^ This was observed in mice from the CCl_4_ group, in which H&E and PSR staining showed significant effects on inflammation and fibrosis induced by CCl_4_. The increase in the levels of AST, ALT, and Hyp noted in the CCl_4_ group indicated that liver injury had occurred and presented a diseased state. The levels of fibrosis makers (*i.e.*, HA, LN, Col IV, and PC III) reflected the formation of HF in the CCl_4_ group. In short, the animal model of HF was successfully established in this study through the use of CCl_4_.^70,71^

Oral administration was selected in this study because it is a non-invasive approach, is associated with high rates of patient compliance, and is of great value for the long-term management of chronic diseases. We used GA to reduce the HF induced by CCl_4_. The results confirmed the *in vivo* and *in vitro* anti-HF effects of GA previously reported in studies.^72,73^ We further confirmed the functional role of RA-GA-NPs on HF. Unlike free GA, RA-GA-NPs demonstrated a remarkable benefit in alleviating the degree of hepatic injury and fibrosis. In H&E staining, this was reflected by the destruction of hepatocytes and significant reduction in the extent of inflammatory cell infiltration. Analysis through PSR staining yielded a similar trend, with a significance reduction in collagen protein expression after the administration of RA-GA-NPs *versus* free GA. In addition, the biochemical levels and fibrosis index were significantly decreased following treatment with RA-GA-NPs *versus* free GA. These results indicated that RA-GA-NPs enhanced the anti-HF effect of GA. The underlying mechanism may explain the application of GA in the NPs polymer matrix. Firstly, the increase in bioavailability was achieved as the NPs have a larger surface area, thereby enhancing the uptake in the gastrointestinal tract. Secondly, a sustained release effect ensured a long circulation time to maintain a sufficient drug concentration for effective anti-fibrotic therapy. Finally, therapeutic delivery strategies were adopted with a specific particle size (*i.e.*, diameter <200 nm) for passive liver targeting and RA binding to RBPR in HSC for active liver targeting. These processes were carried out to maintain high selectivity and specificity for the target.^73–75^ In addition, a significant difference in the suppression of HF production was observed between RA-GA-NPs and GA-NPs. This observation suggested that the integration of RA into BPRP improves the pharmacological effects, as well as the efficacy of active targeted therapy.

## 4 Conclusions

Our data demonstrated that the produced RA-GA-NPs with appropriate physical characteristics, significant controlled release, and positive targeting of HSC could improve the anti-HF effect of GA. We developed an effective, sustained release, and targeted drug therapy, which may contribute to the production of novel DDS for anti-HF therapy. The present findings also suggest that the preparation of RA-GA-NPs is cost-effective and controllable, showing great potential for large-scale industrial production. In summary, the results of this study may provide a new option for the management of HF in clinical trials.

## Author contributions

Yuanguo Xiong and Bing Wu designed the study, conducted experiments, conducted statistical analyses, wrote the article. Xianxi Guo and Dong Shi performed the animal and pathological experiments and collected the data. Hao Xia, Hanlin Xu, and Xiaoxiong Liu supervised the project and edited the final article.

## Conflicts of interest

The authors report no conflicts of interest related to the present study.

## Supplementary Material

RA-013-D2RA07561J-s001
