# Supplementary material for: Galangin delivered by retinoic acid-modified nanoparticles targeted hepatic stellate cells for the treatment of hepatic fibrosis
Source: RSC Adv. 2023 Apr 6;13(16):10987–1001. doi: 10.1039/d2ra07561j (PMC10077338; doi:10.1039/d2ra07561j)
Supplement: RA-013-D2RA07561J-s001 [file RA-013-D2RA07561J-s001.pdf]

Electronic Supplementary Material (ESI) for RSC Advances.
This journal is © The Royal Society of Chemistry 2023

Supplementary materials

The High Performance Liquid Chromatography (HPLC) method had been adopted in detecting the content of galangin (GA) in our study. It had a pump (LC-20AT, Liquid chromatograph, Shimadzu, Japan), a UV detector (SPD-20A, UV/VIS detector, Shimadzu, Japan) measuring at 266 nm, an online degasser, and a Shimadzu Chemstation LC 3D software. The mobile phase used under the chromatographic conditions was developed by mixing acetonitrile(A) and aqueous buffer(B) followed: 0 - 5 min, A: B 25: 75; 5 - 15 min, A: B 55: 45; 15 - 25 min, A: B 65: 35. The aqueous buffer solution had 0.1% phosphoric acid (100: 0.1, v: v). Before use, all the mobile phase solvent was filtered through a 0.45 μm nylon 66 membrane with a Millipore vacuum filtration system. The method was developed using an Agilent extend C18 analytical column (250 × 4.6 mm, 5 μm). The flow rate was 1.0 mL/min, and the column temperature was set at 30 °C. The injection volume was 20 μL, and the wavelength of the detector was 266 nm.

In the HPLC conditions, the GA and the pharmaceutical excipients did not interfere, indicating good specificity of the current method (show in Fig,S1). The linear equation within the concentration range of 0.10 - 4.0 μg/mL was below(SI):

(SI) $A= 51546C - 5448.6 \quad r=0.9992$

Intra- & inter-day precision was within the acceptable deviation of 5%, and the recovery was (96.10 ± 5.98)%, indicating the appropriate method for sample analysis.

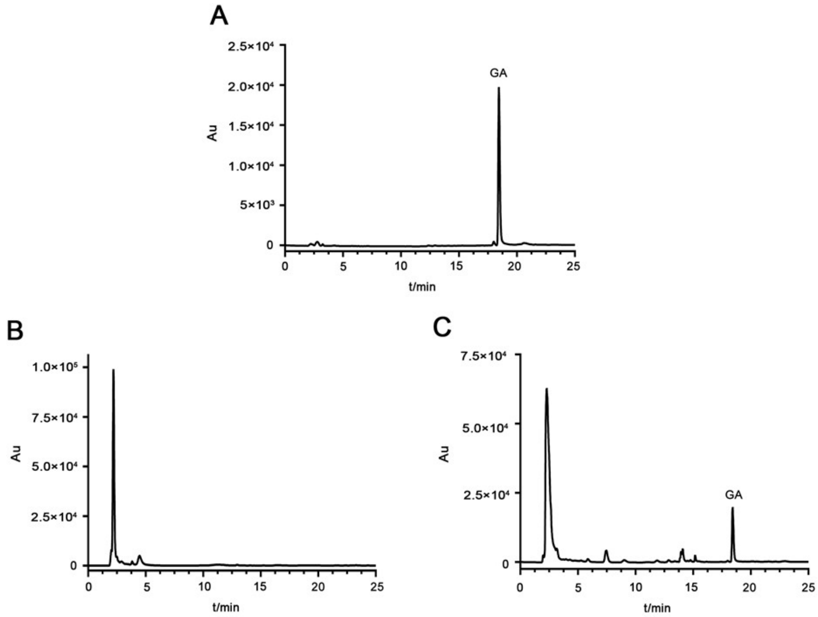

Fig. S1 The plasma HPLC chromatogram of GA-NPs:A, Galangin Control; B. Blank plasma:C, the plasma of GA-NPs.

The stability study showed that the nanoparticles were incubated at 25 °C and 4 °C for six months, characterized by mean particle size, PDI, zeta potential, and encapsulation efficiency at 0, 1, and 6 months, respectively. The result was shown in Fig. S2. When stored at room temperature, the particle size and zeta potential of NPs slightly changed in six months, while the PDI increased significantly. The particle size, PDI, and Zeta potential of nanoparticles showed fewer changes at 4 °C than at room temperature.

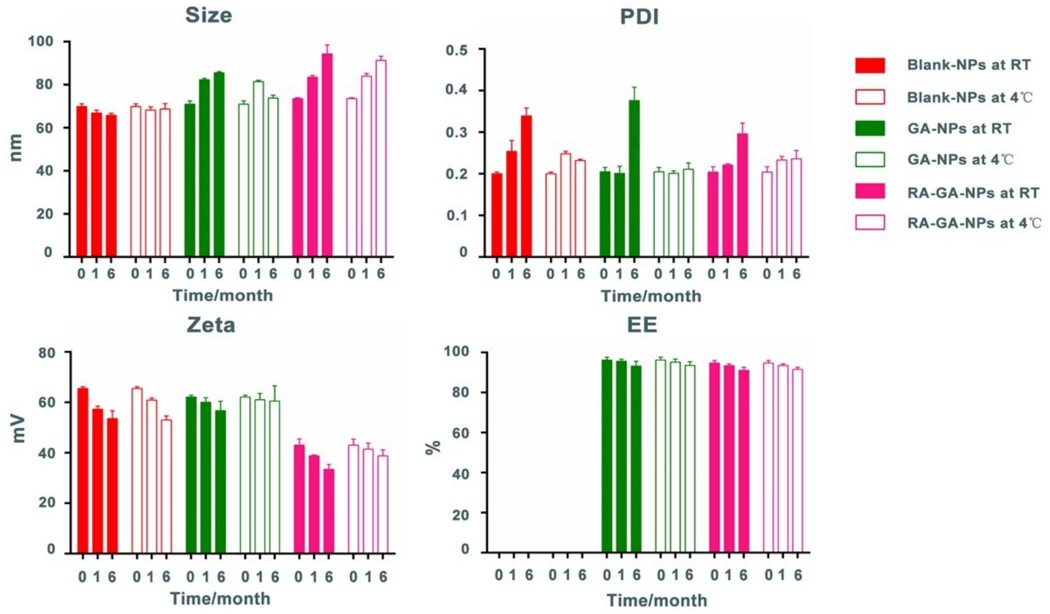

Fig S2. The stability of nanoparticles stored at room temperature (RT) and 4℃ in 6 month, assessed with the particle size, PDI, zeta potential and encapsulation efficiency(EE) (n=3).
